# Implementation Challenges of Remote Cancer Symptom Management With Electronic Patient‑Reported Outcomes in China’s Primary Health Care Settings: Qualitative Study

**DOI:** 10.2196/78333

**Published:** 2025-10-28

**Authors:** Min Li, Jingyu Zhang, Jundi Zheng, Changjin Wu, Zuting Shao, Cheng Lei, Hongfan Yu, Lu Xu, Yu Zhang, Xu Wang, Jin Bai, Qiuling Shi, Xiaojun Dai

**Affiliations:** 1 State Key Laboratory of Ultrasound in Medicine and Engineering Chongqing Medical University Chongqing China; 2 School of Nursing Chongqing Medical University Chongqing China; 3 College of Clinical Traditional Chinese Medicine Yangzhou University Yangzhou China; 4 School of Public Health Chongqing Medical University Chongqing China; 5 Department of Oncology Yangzhou Hospital of Traditional Chinese Medicine Yangzhou China; 6 Department of Thoracic Surgery Sichuan Cancer Hospital & Institute, Sichuan Cancer Center, Affiliated Cancer Hospital of University of Electronic Science and Technology of China Chengdu China; 7 The Affiliated Yangzhou Hospital Nanjing University of Chinese Medicine Yangzhou China

**Keywords:** primary health care settings, electronic patient-reported outcomes, cancer management, implementation science, facilitators, barriers, Consolidated Framework for Implementation Research, Expert Recommendations for Implementing Change

## Abstract

**Background:**

Electronic patient-reported outcomes (ePROs)–based cancer symptom management presents an opportunity to improve patient outcomes by optimizing symptom detection and prompting clinician interventions in tertiary hospitals. However, real-world evidence is limited, especially in primary health care (PHC) settings, which are accompanied by more complex and unknown influencing factors.

**Objective:**

We conducted a qualitative study to identify facilitators and barriers associated with the implementation of ePRO-based symptom management in China’s PHC settings under the implementation science (IS) framework. We further developed strategies and recommendations for real-world practices and health policies.

**Methods:**

This qualitative study was conducted from October to December 2023 in 9 purposively selected PHC institutions (5 urban and 4 rural) across 5 administrative districts of Yangzhou, Jiangsu Province, China. Community-dwelling patients with cancer, PHC providers, and medical supervisors participated in semistructured interviews and focus group discussions. We used 2 subframeworks under the IS framework—the Consolidated Framework for Implementation Research and Expert Recommendations for Implementing Change—to conduct data analysis and generate strategies.

**Results:**

A total of 72 individuals were invited to participate in this study, including 35 community-dwelling patients with cancer (median 66, IQR 60-71.5 years; n=21, 60% men) and 23 PHC personnel (median 45, IQR 27-51 years; n=12, 52.17% men) who participated in semistructured interviews, and 14 medical supervisors (median 47.5, IQR 36.5-54 years; n=10, 71.43% men) who participated in focus group discussions. This study identified 29 barriers and 21 facilitators, and then developed 13 strategies. Crucial challenges include PHC providers’ low self-efficacy and unclear role identification, coupled with community-dwelling patients’ mistrust of primary care, cancer stigma, and fatalistic beliefs, which further reduce motivation; poor integration of ePRO with existing workflows and the absence of performance incentive mechanisms; a lack of nationwide standardized implementation guidelines and quality evaluation criteria; and outdated medical equipment and a limited range of medications. Common challenges included weak collaborative relationships and insufficient funding.

**Conclusions:**

Grounded in the IS framework, our study identifies 3 critical priorities for implementing ePRO-based cancer symptom management in PHC settings, including addressing individual-level motivational deficiencies among community-dwelling patients with cancer and PHC providers by resolving misconceptions, bridging knowledge gaps, and establishing supportive incentives; developing supportive medical partnerships and advancing tiered management systems to empower PHC settings; and creating standardized operational guidelines with clear workflows and implementing real-world data-driven regulatory feedback mechanisms to ensure quality control.

## Introduction

### Background

The increasing global prevalence of cancer, alongside improvements in survival rates, has resulted in an expanding population requiring long-term management of cancer-related symptoms [1-3]. Electronic patient-reported outcomes (ePRO), particularly those with monitoring, alerting, and intervention functions, serve as an effective tool for cancer symptom management [4], with demonstrated benefits including reduced emergency department visits and hospitalizations, as well as enhanced overall survival and health-related quality of life [5-8]. However, this evidence primarily comes from tertiary hospitals, with limited replication in China’s primary health care (PHC) settings [9-11]. To date, with the ongoing advancement of the hierarchical medical system, notable progress has been made in managing cardiovascular diseases and diabetes in China’s PHC settings [12-14]. Yet, cancer management as 1 of the 4 major chronic diseases (cardiovascular and cerebrovascular diseases, cancer, chronic respiratory diseases, and diabetes) in the Healthy China 2030 Action Plan by the State Council—still faces substantial gaps in PHC settings [15-17]. Thus, ePRO-based symptom management could serve as an evidence-based approach to strengthen the core capabilities of PHC institutions [18,19].

The successful and high-quality implementation of ePRO-based symptom management in China’s PHC settings requires careful consideration. Classic studies indicate that translating clinical innovations into practice typically takes 17-20 years, with fewer than 50% achieving widespread adoption [20,21]. Ensuring its integration into real-world practice, therefore, demands research under the framework of implementation science (IS). IS focuses on methods to promote the adoption, integration, and sustained use of evidence-based interventions in real-world settings, recognizing that contextual factors in routine practice—unlike in well-controlled trial environments—are dynamic and can either support or hinder implementation efforts [22-24]. A critical step in the preimplementation phase of IS is identifying barriers and facilitators to uptake through active engagement with all stakeholders [25,26].

### Objectives

Therefore, we conducted a qualitative study to identify facilitators and barriers associated with implementing ePRO-based symptom management in China’s PHC settings under the IS framework, involving key stakeholders: community-dwelling patients with cancer, PHC providers, and medical supervisors. Based on these findings, we developed strategies and recommendations for clinical practice and health policies to support the implementation of ePRO-based cancer symptom management in China’s PHC settings.

## Methods

### Study Design and Sites

We purposively selected 5 administrative divisions of Yangzhou City, Jiangsu Province—Baoying County, Jiangdu District, Yizheng City, Hanjiang District, and Gaoyou City—that cover the high, medium, and low economic levels of Yangzhou, thereby ensuring social and economic diversity. From these divisions, we further purposively selected 9 PHC institutions, of which 5 are urban and 4 are rural. Within these 9 sites, purposive sampling was used to select participants who were eligible and could provide rich insights into the research question. A completed Standards for Reporting Qualitative Research checklist is provided [27].

### Study Participant Sampling and Recruitment

Study participants included PHC providers (doctors and nurses from local PHC institutions or oncology departments), medical supervisors (staff from local health commissions or bureaus and Centers for Disease Control and Prevention), and community-dwelling patients with cancer. PHC providers and supervisors were eligible if they had worked in their organizations for more than 3 months, were responsible for providing professional support or PHC for patients with cancer, and were willing to sign informed consent. Community-dwelling patients with cancer aged ≥18 years were eligible if they resided in the community under the jurisdiction of the selected PHC institutions and were willing to provide informed consent; those with severe medical conditions unsuitable for interviews were excluded. One week before the interviews, we identified 20 community-dwelling patients with cancer and 20 PHC providers as interviewees via lists from 9 PHC institutions. One day prior, all were successfully contacted by telephone; only 1 community-dwelling patient with cancer and 2 PHC providers explicitly stated their inability to participate. On the interview day, 1 community-dwelling patient with cancer withdrew during the process, while all the other participants completed it. Similarly, for the second round, among 20 community-dwelling patients with cancer and 6 PHC providers identified, 2 community-dwelling patients with cancer declined to participate. On its day, 1 community-dwelling patient with cancer and 1 PHC provider failed to attend due to schedule delays, and the rest completed the interviews smoothly. Meeting invitations were sent 15 days before the focus group discussion, and all 14 scheduled participants attended.

### Data Collection

Semistructured interviews were conducted with PHC providers and community-dwelling patients with cancer. Interviews were scheduled in advance by a single interviewer at a mutually agreed time and place to ensure privacy, allowing participants to share views freely. A standard operating procedure (SOP) for interviews was developed beforehand (Multimedia Appendix 1). The initial interview guide was conducted with 18 community-dwelling patients with cancer and 18 PHC providers between September and October 2023 (Multimedia Appendix 2). After analyzing data from the first round of interviews, the guide was revised, and the remaining participants were interviewed in November 2023 (Multimedia Appendix 3). Fourteen medical supervisors participated in focus group discussions in December 2023, with the discussion guide developed based on semistructured interview results (Multimedia Appendix 4). All interviews and discussions were conducted face-to-face in Mandarin. After each interview, researchers summarized findings and invited participants to verify key information to avoid misunderstandings and enhance study credibility. Sample size was determined by the principle of code saturation, defined as the point at which no new issues are identified and the codebook stabilizes [28]. Interviews were initially conducted with 32 community-dwelling patients with cancer and 20 PHC providers. As analysis progressed, themes reached saturation with no new themes or relationships emerging. Three additional community-dwelling patients with cancer or PHC providers were included to further validate extracted themes. All procedures adhered to informed consent and confidentiality principles. Written informed consent, including permission for audio recording, was obtained before conducting interviews and discussions. Participants were informed they could refuse questions or withdraw from this study at any time. Participant identifiers were anonymized using alphanumeric codes (P1-P35 for community-dwelling patients with cancer; D1-D23 for PHC providers; and M1-M14 for medical supervisors) throughout this study.

### Conceptual Framework

We applied the Consolidated Framework for Implementation Research (CFIR) 2.0 to guide study analysis and the Expert Recommendations for Implementing Change (ERIC) to generate strategies [29,30]. CFIR 2.0 includes 47 constructs across 5 domains, providing a practical framework for systematically assessing potential barriers and facilitators [31]. We used CFIR to conduct a preimplementation assessment of ePRO in China’s PHC settings and characterize feasibility and acceptability from the stakeholders’ perspectives. The CFIR-ERIC Matching Tool compiles 73 implementation strategies based on survey responses from 169 “implementation experts,” grouped into 9 clusters by strategy type. The ERIC list includes “level 1” strategies (endorsed by >50% of experts as top 7 for a barrier) and “level 2” strategies (endorsed by 20%-50% of experts) [30]. Per literature, “level 1” strategies with higher expert consensus are recommended as primary options, while “level 2” strategies should be considered if more applicable to the context [32,33]. We used CFIR-ERIC to generate potential strategies for identified barriers, then prioritized them by importance or urgency.

### Data Analysis

All interviews and discussions were audio-recorded and transcribed verbatim. Alphanumeric codes were used as participant identifiers throughout analysis to maintain confidentiality, with data access restricted to research team members. Analysis followed directed content analysis, an inductive-deductive approach guided by existing knowledge about the phenomenon or relevant theory. First, 2 researchers independently reviewed transcripts iteratively to immerse themselves in the data and inductively generate preliminary codes for influencing factors around emergent concepts. Second, similar preliminary codes were grouped into themes and deductively mapped to CFIR 2.0. Coding discrepancies were resolved through team discussion to optimize intercoder reliability. All quotations were translated into English via forward-backward translation to ensure rigor, with each citation marked by participant role and study number to avoid identification. Data coding and analysis were conducted in Chinese using NVivo (version 12; QSR International) from October 2023 to October 2024. Identified barriers were mapped to the CFIR-ERIC Matching Tool to obtain potential implementation strategies and their cumulative percentages. “Level 1” strategies and the top 5 cumulative percent ERIC strategies were selected to ensure inclusion of critical, highly applicable theory-informed strategies. To enhance contextual relevance, stakeholder focus groups adapted these strategies to the practical realities of ePRO adoption in Chinese PHC settings. Descriptive statistics for quantitative data (mean, SD, median, and IQR) were calculated using the Statistical Analysis System (version 9.4) to summarize participant demographic characteristics.

### Ethical Considerations

Ethics approval and consent to participate: ethical approval for the process evaluation was granted by the Ethics Committee of the Yangzhou Hospital of Traditional Chinese Medicine (identifier: ChiCTR2200065569). Participants were informed of their right to refuse questions or withdraw from this study at any time, and were asked to provide written informed consent that details this study’s purpose, as well as the nature, use, and management of their data. Throughout this study, participant identifiers were anonymized using alphanumeric codes to ensure no individual could be identified in this paper’s content or supplementary materials. No compensation was offered to participants for their participation.

## Results

### Overview

**Figure 1:**
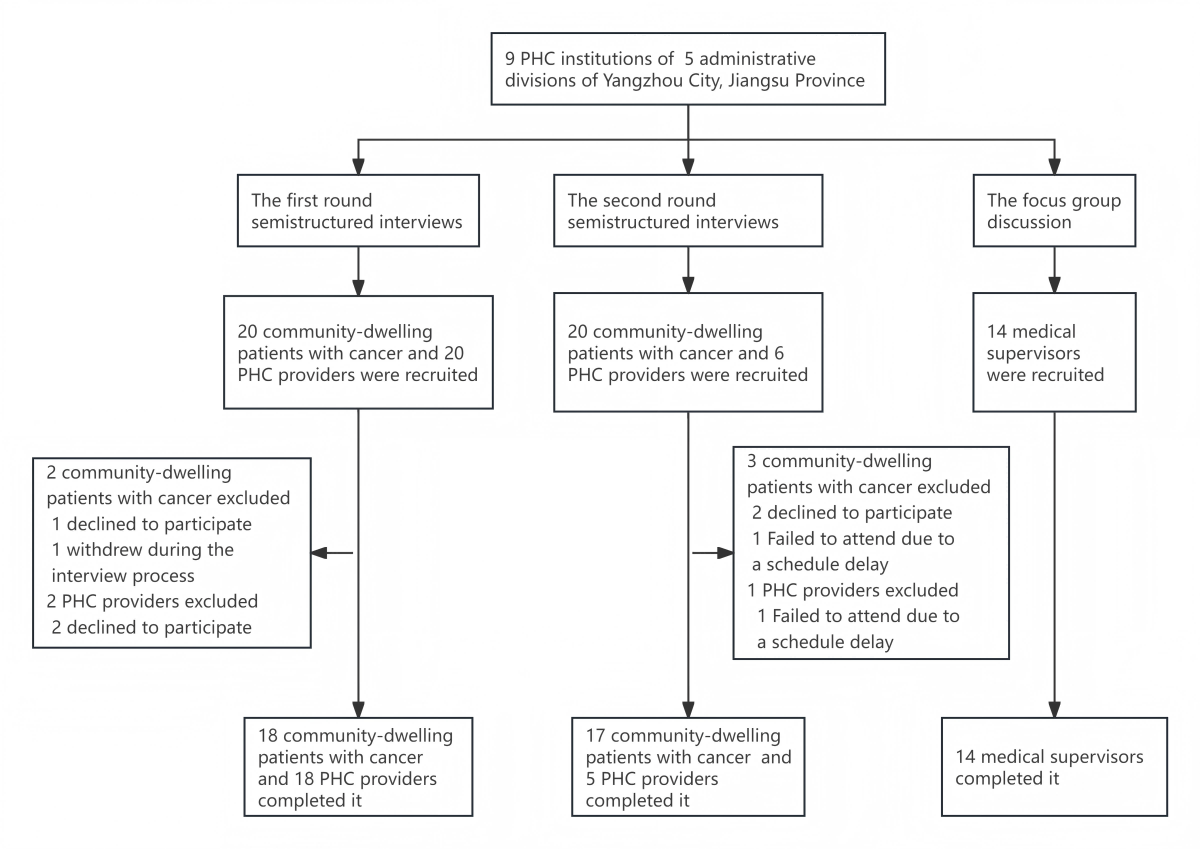
Participant recruitment flow in the qualitative study. PHC: primary health care.

In total, 72 participants were invited to participate in 58 semistructured individual interviews and 1 focus group discussion (Figure 1). Semistructured interviews included 35 community-dwelling patients with cancer (median 66, IQR 11.5 years; n=21, 60% men) and 23 PHC personnel (median 45, IQR 24 years; n=12, 52.17% men). The focus group discussion included 14 medical supervisors (median 47.5, IQR 17.5 years; n=10, 71.43% men). Around 47.83% (n=11) of PHC personnel and 92.86% (n=13) of medical supervisors had 10-36 years of medical-related work experience. The mean interview length was 20.86 (SD 4.93) minutes for community-dwelling patients with cancer and 23.69 (SD 6.49) minutes for PHC providers, with both excluding the introductory part of the interview (Table 1).

**Table 1:** Demographic characteristics of the participants.

Demographic characteristic			In-depth interview					Focus group discussion
			Community-dwelling patients with cancer (n=35)		Primary health care provider (n=23)		Medical supervisor (n=14)	
**Gender, n (%)**								
	Men	21 (60)		12 (52.17)		10 (71.43)		
	Women	14 (40)		11 (47.83)		4 (28.57)		
Age (years), median (IQR)			66 (60-71.5)		45 (27-51)		47.5 (36.5-54)	
Interview length (minutes), mean (SD)			20.86 (4.93)		23.69 (6.49)		N/A^a^	
**Cancer category, n (%)**								
	Respiratory system	8 (20)		N/A		N/A		
	Digestive system	19 (17.14)		N/A		N/A		
	Endocrine system	5 (14.29)		N/A		N/A		
	Reproductive system	2 (2.86)		N/A		N/A		
	Urinary system	1 (2.86)		N/A		N/A		
**Duration of illness (years), n (%)**								
	1-3	25 (71.43)		N/A		N/A		
	4-10	10 (28.57)		N/A		N/A		
**Medical-related work (years), n (%)**								
	0-3	N/A		8 (34.78)		0 (0)		
	4-10	N/A		4 (17.39)		1 (7.14)		
	10-36	N/A		11 (47.83)		13 (92.86)		
**Professional title, n (%)**								
	Junior	N/A		11 (47.83)		0 (0)		
	Intermediate	N/A		5 (21.74)		7 (50)		
	Senior	N/A		7 (30.43)		7 (50)		

^a^N/A: not applicable.

We identified 50 factors across 4 CFIR 2.0 domains that impacted remote cancer symptom management with ePRO in China’s PHC settings, consisting of 29 barriers and 21 facilitators (detailed by domain in Multimedia Appendix 5). A total of 13 theory-informed implementation strategies were selected, including 8 “level 1” strategies and the top 5 strategies by cumulative percentage. They were then optimized into context-adapted specific strategies through 2 rounds of group discussions (Multimedia Appendices 6 and 7).

### Innovation

#### Acknowledged Advantages of ePRO

A total of 7 facilitators and 4 barriers were identified in the 8 constructs of the innovation domain within CFIR 2.0 (Multimedia Appendix 5). Some medical supervisors and senior oncology clinicians, familiar with ePRO through reviewing published literature, expressed support for its substantial advantages in cancer symptom management compared to routine approaches (eg, survival follow-up). As a symptom management tool within medical devices, ePRO demonstrated strong product design, with digital features facilitating implementation. Consensus was reached regarding its adaptability and testability in PHC settings.

I’ve known that several top-tier hospitals’ oncology departments have already rolled out this digital technology for comprehensive symptom management, and the results have been really positive. They’ve even published articles in leading international journals, with short-term effectiveness and long-term outcomes. However, we just call up community-dwelling cancer patients to collect some information, fill out forms. If we could gradually pilot ePRO here, tweaking it to fit our local environment and population, it could help grassroots village doctors better manage these patients’ symptoms.

Medical supervisor 4 (PHC Institution)

#### Operation Complexity of ePRO

Implementing new technology presented challenges, particularly for older village doctors. For PHC providers, managing symptoms of community-dwelling patients with cancer was more complex than routine survival follow-up, involving a broader range of tasks. When the symptom scores of a community-dwelling patient with cancer reached alert thresholds, PHC providers had to promptly assess the patient’s condition status via phone or in-person, deliver interventions (eg, education, psychological support, medication, and referrals), and continuously monitor progress. From the perspectives of community-dwelling patients with cancer, weekly symptom reporting via mobile devices was also challenging, especially for older adults.

What I’m most worried about is the extra workload once we start using it. We’re already short-staffed at the grassroots level. Plus, a lot of our village doctors are older, in their 60s or 70s, and might struggle to pick up new skills. I hope we can fold it into our current workflow and keep the process simple.

Medical supervisor 6 (PHC Institution)

That thing you mentioned—ePRO—it sounds hard to use. I’m getting on in years, my eyes aren’t great, and I don’t use my phone much.

Community-dwelling patient with cancer 3

#### Supervision Complexity of ePRO

Medical supervisors from the Health Commission noted that ePRO coverage spanned PHC systems to top-tier hospitals, making safety and quality supervision across the service pathway challenging. These requirements demanded ongoing investment in human, material, and financial resources. A key advantage for community-dwelling patients with cancer, however, was that services were free during the trial phase.

Honestly, rolling out ePRO at the Health Commission level isn’t easy, which means coordinating across multiple levels of institutions. And we have to make sure the quality is there. If we’re going to do this, we need to do it right. It’s good for patients, though—they won’t pay a cent until it’s officially adopted. And if we manage their symptoms well, it could even lighten their financial load.

Medical supervisor 7 (Health Commission)

### Outer Setting

#### Implementation of ePRO Is Needed

A total of 5 facilitators and 5 barriers were identified in the 5 constructs of the outer setting domain within CFIR 2.0 (Multimedia Appendix 5). Many PHC providers noted that patients with cancer increasingly returned to the community post discharge but received little supervision. Effective management was essential to alleviate symptoms and improve quality of life, aligning with Healthy China 2030 goals and hierarchical medical system policies.

In the past, people heard “cancer” and thought “death.” But now, it’s more like a chronic disease—both incidence and survival rates are up. Cancer patients spend most of their time in the community except during active hospital treatment, so primary-level management is key. It directly affects their quality of life and how long they live.

Medical supervisor 2 (PHC Settings)

#### Limited Outer Setting

Unlike the National Essential Public Health Service Package (eg, hypertension, diabetes, and fall prevention), this initiative faced barriers: a lack of standardized care pathways, evaluation metrics, and assessment criteria for ePRO; weak collaboration between PHC settings and tertiary hospitals; unintegrated electronic medical records hindering digital implementation; and insufficient financial support, with no dedicated subsidies for community-dwelling patients with cancer.

We handle diabetes and hypertension pretty well at the PHC level because there’s dedicated staff for that. When higher-ups set mandatory targets, with end-of-year assessments tied to salaries, things get done quickly. But cancer management? It’s just encouraged, not required—no hard targets. And we’re not sure how to do it right. Is there a standardized process like there is for diabetes or hypertension? Plus, patients don’t get any financial help, which makes it even tougher.

Medical supervisor 11 (PHC Settings)

We don’t collaborate with higher-level hospitals, so we can’t access patients’ treatment records from there. Going into patients’ homes without that information often leads to misunderstandings or conflicts. It makes management really tough.

PHC provider 3

### Inner Setting

#### PHC Settings Have Been Progressively Enhanced

A total of 11 barriers and 7 facilitators were identified in 14 constructs of the inner setting within CFIR 2.0 (Multimedia Appendix 5). Most medical supervisors agreed on advocating for ePRO implementation. With national investments in PHC services, the workforce had become more professionalized, including specialized teams in clinical diagnosis or treatment, nursing, public health, and management, collaborating to handle grassroots tasks. Through information-based management of hypertension, diabetes, severe mental illness, and cancer survivorship follow-up, basic digital capabilities had been established. Additionally, government-sponsored continuing education programs steadily improved grassroots personnel’s expertise.

I think we’re capable of taking on cancer management and trying out this new technology (ePRO). Right now, we have family doctor teams at the grassroots level—clinical doctors, nurses, public health workers, and village doctors. Sometimes, we even get support from higher-level hospital experts. We earn continuing education credits every year, so our skills are improving. For community-dwelling cancer patients, we need to quickly build a personalized service model that fits their needs.

PHC provider 5

#### Concerns About ePRO’s Goals and Meaning

Some grassroots doctors lacked clear alignment with the overall goals of ePRO-based symptom management, and their value alignment with this initiative required further strengthening. They expressed concerns about PHC institutions’ capacity to manage geographically dispersed community-dwelling patients with cancer, especially in low-density rural areas. Additionally, effective collaboration with higher hospitals required new operational pathways and dedicated staff, which would be hard to integrate into existing workflows. Undoubtedly, cancer management posed greater challenges than hypertension or type 2 diabetes care.

I’m not very familiar with this new technology—ePRO, our community health station covers all residents here—chronic diseases, kids, expectant mothers. Every PHC provider is already swamped: monthly assessments for hypertension and diabetes patients, maternal and child care. We can’t start on cancer management until we finish those tasks first.

PHC provider 12

We’re busy every day, told it’s for the people, serving the community—but what’s the end goal? No one knows. It feels like we’re always rushing, but none of it seems to mean much.

PHC provider 19

#### Inadequately Available Resources in PHC Settings

PHC settings faced resource limitations. Financially, subsidies per PHC institution were markedly lower than those for tertiary hospitals. PHC institutions also had outdated, limited-function medical devices and incomplete medication selections, undermining cancer management quality. PHC personnel noted insufficient oncology-focused continuing education, which was often examination-oriented—primarily for annual assessments and professional title evaluations.

From our grassroots perspective, the main problem is funding, which leads to outdated equipment. Our community hospitals are missing a lot of basic devices, so we can’t run essential tests for cancer patients, and we’re short on medications. People ask, “What can you actually do here?” I think with more funding, we could do so much more—and do it better.

PHC provider 16

### Characteristics of Individuals

#### Low Motivation and Opportunity for PHC Providers

**Figure 2:**
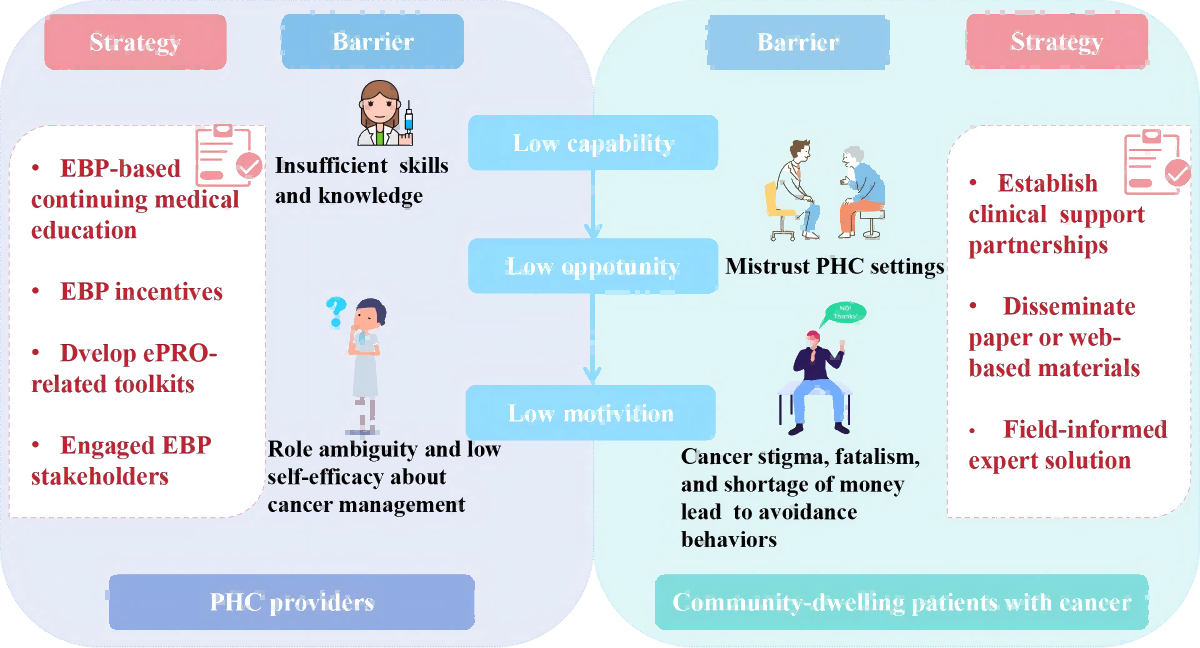
Motivational deficits in ePRO implementation among PHC providers and community-dwelling patients with cancer: influencing factors and formation mechanisms within the COM-B model. COM-B: Capability, Opportunity, Motivation, Behavior; EBP: evidence-based practice; ePRO: electronic patient-reported outcomes; PHC: primary health care.

A total of 9 barriers and 2 facilitators were identified in the 4 constructs of the Characteristics of Individuals domain within CFIR 2.0 (Multimedia Appendix 5). Among these barriers, direct influential relationships were found, based on the Capability, Opportunity, Motivation, Behavior (COM-B) model (Figure 2). PHC providers showed little need to implement ePRO for community-dwelling patients with cancer, as it was nonmandatory and offered few benefits due to unclear performance incentives. They also felt they lacked the knowledge and skills to use ePRO for cancer management. Compounding this, limited opportunities arose because community-dwelling patients with cancer distrusted PHC providers—perceiving them as less competent—and avoided PHC-led cancer management. Consequently, PHC providers had low motivation, unclear roles in ePRO, and even doubted their ability to contribute, viewing cancer care as the responsibility of tertiary hospitals. A summary of selected key quotations is provided in Textbox 1.

**An overview of the quotes from primary health care (PHC) providers we find the most relevant or noteworthy.**

- “If this new ePRO technology just adds to our workload without any incentives, we (PHC providers) won’t be motivated—we all have to make a living. Our community’s cancer management is already weak, and our professional knowledge is limited. A lot of community-dwelling cancer patients skip us entirely and go straight to top hospital doctors.” [PHC provider 20]
- “If we’re doing all this for free, where’s our income coming from? Managing cancer patients isn’t even our main job here. We’re short on staff and equipment—our CT scanners can’t compare to top hospitals’. Patients rarely choose us for cancer care. Even if we invite them, they won’t come. Our equipment’s outdated, and we just don’t have the right medications.” [PHC provider 8]
- “Right now, I’m not really sure what we (PHC providers) are supposed to do in this ePRO project, or if we can even do it well. If there were clear role assignments and reference guides, we could just follow the steps.” [PHC provider 19]

#### Cancer Stigma and Fatalism Among Community-Dwelling Patients With Cancer

Most community-dwelling patients with cancer lacked access to information on treatment, rehabilitation, and financial assistance policies. They also had limited trust in PHC institutions’ diagnostic and treatment capabilities, leading them to seek care directly at tertiary hospitals for any discomfort, increasing their own time and financial burdens. Conversely, some community-dwelling patients with cancer adopted passive or neglectful attitudes toward treatment due to financial hardship, cultural beliefs, or fatalism. Others avoided PHC providers due to cancer stigma, fearing others would learn of their condition. Thus, despite needing standardized symptom management, these patients often struggled to seek or receive support. A summary of selected key quotations is provided in Textbox 2.

**An overview of the quotes from respondents we find the most relevant or noteworthy.**

- “Some cancer patients want to keep their illness private, especially in the community. When we follow up, they won’t admit they have cancer, so they won’t accept any services.” [primary health care (PHC) provider 1]
- “We don’t know much about this ePRO thing. Community doctors’ check-ups aren’t helpful—they don’t have the right equipment, so they can’t find anything. I’d rather just contact my cancer surgeon directly.” [Community-dwelling patient with cancer 29]
- “I won’t go to a community hospital for cancer. They don’t have my medical records—can’t check my condition on their computer (electronic medical record system). They’re not specialists in cancer treatment. I’d rather ask an expert.” [Community-dwelling patient with cancer 14]
- “We’re old. We don’t want to burden our kids—with costs or time. We don’t want to burden the country. Cancer makes us different from healthy people. But no one’s looking out for us. I walk alone now to get exercise. After all, I’m not normal anymore. With cancer… how much time I have left—it’s all up to fate.” [Community-dwelling patient with cancer 5]

#### Strategy Design

**Figure 3:**
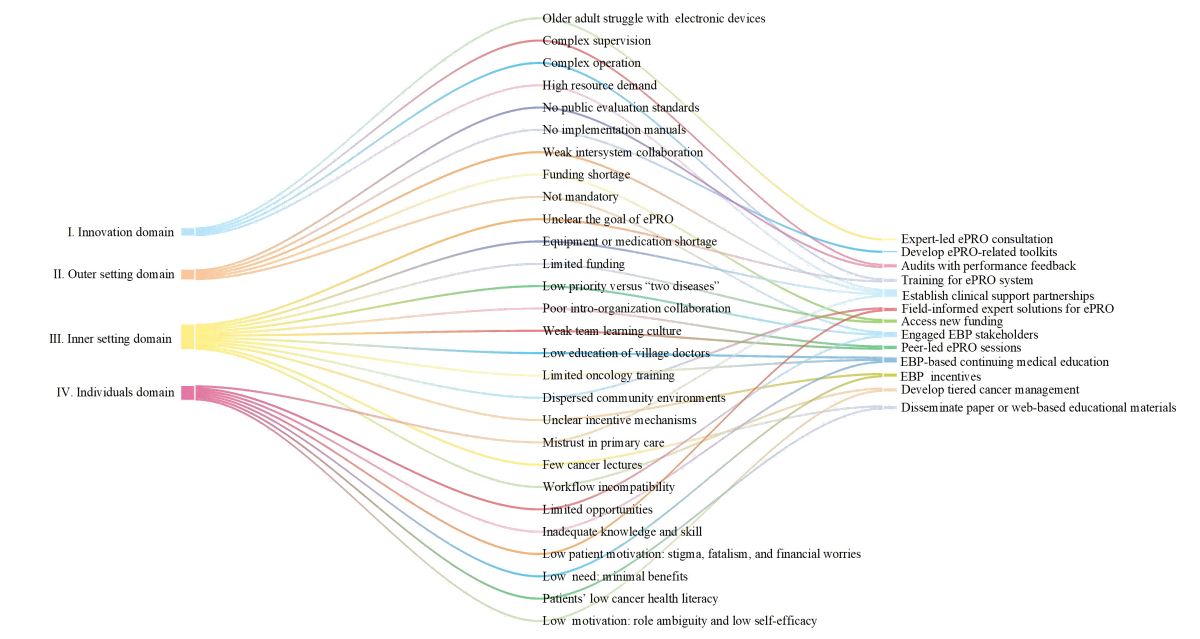
Barriers and matched implementation strategies for the implementation of ePRO-based cancer symptom management in China’s PHC settings. EBP: evidence-based practice; ePRO: electronic patient-reported outcomes; PHC: primary health care.

We developed 13 potential strategies from the ERIC list to address identified barriers (Figure 3). These strategies were refined through 2 rounds to fit the context of ePRO implementation in PHC settings (Multimedia Appendix 7). First, engaging evidence-based practice (EBP) stakeholders is expected to improve the implementation environment and organizational culture. Establishing clinical support partnerships and tiered cancer management approaches will help to alleviate equipment or medication shortages and information silos. Developing ePRO toolkits, providing system training, offering field-informed expert solutions, and conducting peer-led and expert-led ePRO consultations could streamline workflows and resolve practical obstacles. Disseminating paper or web-based educational materials can effectively improve the health literacy of community-dwelling patients with cancer; additionally, carrying out EBP-based continuing medical education (CME) will substantially enhance the competence of PHC providers in cancer diagnosis and treatment knowledge and skills. Securing new funding and offering EBP incentives will address financial and motivational issues. Finally, implementing audits with performance feedback will help ensure the quality of management.

## Discussion

### Principal Findings

This qualitative study comprehensively assessed challenges in implementing ePRO-based symptom management in PHC settings from the perspectives of community-dwelling patients with cancer, PHC providers, and medical supervisors. The innovative technology requires sufficient learning time, particularly for older village doctors and older adult community-dwelling patients with cancer. Due to its nonmandatory nature, nationwide standardized implementation and quality evaluation criteria are lacking. At the institutional level, poor integration of ePRO with existing workflows led to low prioritization and absent performance incentives. Some PHC providers also had a limited understanding of program objectives. Outdated equipment and limited medications in PHC settings further constrained effective management. At the individual level, PHC providers’ low self-efficacy and unclear roles, coupled with mistrust, cancer stigma, and fatalism, reduced motivation bilaterally by community-dwelling patients with cancer. Common challenges included weak intersystem collaboration and insufficient funding. This study also developed 13 strategies to address these issues. The individual characteristics domain contained critical, easily overlooked barriers: low motivation among community-dwelling patients with cancer and PHC providers impeded implementation at the grassroots level. The COM-B model posits that behavior stems from sufficient capability, opportunity, and motivation [34]. Many Chinese grassroots doctors have limited education—some are underqualified—leading to uneven clinical competencies [35]. While China’s commitment to strengthening the PHC system through the National Essential Public Health Service Package has improved its ability to manage chronic conditions such as diabetes and hypertension [36], its cancer management capacity remains constrained by the specialized expertise required [37,38]. Of nationwide reforms to CME programs for health care professionals, these initiatives have largely devolved into mere credit-earning exercises [36,38]. This situation underscores the urgent need to develop substantive, practice-oriented CME programs led by experienced clinical specialists, with the ultimate goal to enhance PHC providers’ skills in cancer symptom identification and management. Our study also found that PHC providers face limited opportunities to implement cancer symptom management. Due to the mistrust and misconceptions of community-dwelling patients with cancer [36], they tend to bypass village clinics or community health centers for tertiary hospitals, further eroding PHC providers’ self-efficacy and motivation [39]. Establishing clinical support partnerships—for example, having renowned specialists hold mentoring clinics at PHC institutions—shows community doctors they are not working alone but are supported by higher-level teams, enabling better tiered symptom management [40]. Evidently, the implementation of this strategy necessitates close and stable collaboration across health care tiers.

Younger community-dwelling patients with cancer often experience strong cancer stigma, leading to restricted communication, delayed medical help-seeking, refusal of follow-up care, and poor treatment adherence [41,42]. This challenges PHC providers but not tertiary oncologists, whose patients actively seek help, and their interactions take place in distant hospitals, which seems to be a safer environment for them. We also found that most older adult community-dwelling patients with cancer hold fatalistic views about cancer, with pessimistic attitudes toward treatment and symptom management [43]—often rooted in limited knowledge and superstitious and rigid culture. The community-dwelling patients with cancer also feared burdening families, financially or emotionally, leading to social withdrawal. These issues comprehensively hinder their engagement in ePRO-based cancer symptom management. These specific issues require targeted solutions—such as peer-led sessions and expert consultations—that are needed, including protecting the health information of community-dwelling patients with cancer [44] and providing services via acceptable methods such as ePRO with digital or remote characteristics [45]. All measures require explicit trust and informed consent from both community-dwelling patients with cancer and their families. Moreover, improving living environments of community-dwelling patients with cancer through enhanced family support and socioeconomic assistance [46], including disseminating paper or web-based education for community-dwelling patients with cancer and their families, are particularly vital. Education lectures should cover cancer knowledge, new symptom management technologies, PHC advancements, and national support policies. Developing such materials demands time and effort to gather up-to-date medical and policy information and translate it into accessible content—simply revising existing materials is insufficient [47]. Notably, some PHC providers took passive attitudes toward cancer management, failing to recognize it as part of their health care responsibility—consistent with prior findings [48,49]. While others embraced innovation, they remained confused about their roles and responsibilities. Ambiguity in PHC providers’ roles in cancer care is a commonly reported implementation barrier [50,51]. Unlike patients with “two chronic conditions” (diabetes and hypertension) managed under capitation fees [52], patients with cancer lack similar incentives, leaving PHC workers worried about increased workload [11,37]. This nonmandatory status lowers the priority of digital cancer management in PHC settings. Addressing this requires first engaging influential multi-stakeholder advocates with professional leadership and social mobilization capacity [53]. The next is training PHC providers to clarify ePRO system understanding and role expectations, paired with internal incentives tied to role-specific responsibilities [54].

Three inevitable obstacles exist in the outer setting domain of a critical barrier that stems from the insufficient collaboration between PHC and tertiary hospitals—a global systemic challenge, particularly in China, where PHC institutions have neither become the natural first point of contact for patients nor the established genuine coordination mechanisms with specialty care [11,35]. Fragmented patient information sharing, intrinsically linked to poor coordination, may be a direct operational consequence. These issues leave PHC providers working in silos, lacking efficient pathways to tertiary oncologists, and compromising care quality [50,54]. To address the impasse, the Chinese Government issued a tiered health care system requiring tertiary, secondary, and primary facilities to operate within designated roles while integrating care coordination and bidirectional referrals through medical alliances [55,56]. However, scaling these alliances has been slow, hindered by limited incentives and concrete policies [52,57]. This highlights the need to secure national public funding to address insufficient financial resources. Beyond funding, establishing standardized use mechanisms and transparent incentives is critical to building stakeholder consensus [54]. These steps will enable a tiered interinstitutional collaboration system, resolving coordination gaps and information silos to achieve stratified cancer symptom management.

Three additional factors reflected innovation and inner setting domains. As an innovative practice, ePRO-based cancer symptom management with PHC providers as gatekeepers faces multidimensional challenges compared to conventional survival follow-up and data registry systems [58,59]. Recommended steps include on-site assessments to identify PHC providers and community-dwelling patients with cancer needs for device operation, collaborating with information technology specialists for iterative testing to optimize usability (eg, reducing unnecessary steps and simplifying interfaces) [60], and developing ePRO toolkits or manuals to streamline daily use—with sufficient training and reference materials. Beyond device operation, ePRO-based symptom management involves alert handling and medical intervention, demanding higher operational intensity and professional complexity than chronic disease management [61,62]. PHC providers generally lack relevant experience, while equipment and medication supplies are inadequate [63]. Establishing symptom-specific SOPs is essential to enable PHC providers to rapidly manage warning symptoms, facilitate timely referrals, and fully leverage tiered cancer management systems with collaborative support mechanisms. The development of these symptom-specific SOPs must integrate international guidelines with local data through expert consensus to maintain professional authority [9]. As the first ePRO pilot in China’s PHC settings involving multi-institutional collaboration [64], standardized quality evaluation protocols are lacking. Beyond establishing quality assessment metrics and standards, we recommend optimizing supervision through a real-world data-driven regulatory feedback mechanism, including monthly quality control assessments of follow-up adherence, longitudinal symptom trends, and triggered clinical alerts [65].

### Implication for Future Research

This study enhances our understanding of the real-world implementation environment of ePRO in the PHC settings, guided by IS theory. This study identifies barriers and facilitators while developing actionable implementation strategies, contrasting with the efficacy outcomes emphasized in randomized controlled trials. Based on our results, we suggest that future research should prioritize the implementation of multidimensional ePRO-based intervention strategies in real-world PHC settings, using longitudinal study designs to dynamically evaluate the entire implementation process. Key evaluation metrics should encompass operational efficacy parameters, including temporal costs, human resource allocation, and economic expenditures. The ultimate dual objectives are (1) to deliver substantive benefits to all stakeholders, and (2) to achieve seamless integration of ePRO systems into routine clinical workflows.

### Strengths and Limitations

This is the first study investigating real-world implementation challenges of digital therapeutics for cancer symptom management via ePROs in China’s PHC settings. It engaged all critical stakeholders: community-dwelling patients with cancer, PHC providers, health care administrators, tertiary oncologists, and provincial or municipal regulators. Based on the IS framework, we used the CFIR to systematically map multidimensional barriers across 4 domains. Finally, through the ERIC and 2 rounds of group discussions, context-specific and actionable strategies were codeveloped, ensuring scientific validity and contextual fidelity to China’s PHC reality.

Limitations include this study’s exclusive focus on Yangzhou, Jiangsu Province—generalizability to other Chinese regions with varying economic and health care resource levels requires caution. The readers should consider contextual details, such as whether the pilot communities are urban or rural, the population size of the jurisdiction, whether the economic conditions are roughly matched, and the differences in health care systems. Second, while purposive sampling may introduce selection bias, efforts ensured sample representativeness and richness. Five Yangzhou administrative regions spanning high-, medium-, and low-economic levels were selected, plus 9 primary health institutions (5 urban and 4 rural). Respondent sampling targeted 2 groups: medical personnel with primary-level experience and cancer management expertise (including general practitioners, nurses, and managers); and community-dwelling patients with cancer, with diverse cancer types and stages considered. Third, factors were identified qualitatively without causal inference; further quantitative surveys are needed to confirm findings.

### Conclusions

Under the Healthy China 2030 framework, promoting ePRO-based cancer symptom management in PHC settings is of great policy relevance and practical value. This innovation requires first systematically analyzing the real-world implementation environment, then developing targeted, adaptable, context-specific strategies. Grounded in the IS framework, our study identifies 3 critical priorities: addressing individual-level motivational deficiencies among community-dwelling patients with cancer and PHC providers by resolving misconceptions, bridging knowledge gaps, and establishing supportive incentives; developing supportive medical partnerships and advancing tiered management systems to empower PHC settings; and creating standardized operational guidelines with clear workflows and implementing real-world data-driven regulatory feedback mechanisms to ensure quality control.

## Data Availability

Requests for data access will be reviewed by the corresponding authors to ensure that the use of data is in line with the terms of ethics approvals and principles. Access to data will be made available following publication upon reasonable request.

## Appendix

Multimedia Appendix 1 Standard operating procedure of the semistructured interview.

Multimedia Appendix 2 Semistructured interview guide (I version).

Multimedia Appendix 3 Semistructured interview guide (Ⅱ version).

Multimedia Appendix 4 Expert panel discussion outline.

Multimedia Appendix 5 Identified facilitators and barriers in the 4 domains of the CFIR framework. CFIR: Consolidated Framework for Implementation Research.

Multimedia Appendix 6 Implementation strategies as recommended by the CFIR-ERIC matching tool. CFIR-ERIC: Consolidated Framework for Implementation Research–Expert Recommendations for Implementing Change.

Multimedia Appendix 7 The process of optimizing 13 original strategies into context-adapted specific strategies through 2 rounds of group discussions.

## Abbreviations

CFIR: Consolidated Framework for Implementation Research
CME: continuing medical education
COM-B: Capability, Opportunity, Motivation, Behavior
EBP: evidence-based practice
ePRO: electronic patient-reported outcome
ERIC: Expert Recommendations for Implementing Change
IS: implementation science
PHC: primary health care
SOP: standard operating procedure
