# Supplementary material for: Implementation Challenges of Remote Cancer Symptom Management With Electronic Patient‑Reported Outcomes in China’s Primary Health Care Settings: Qualitative Study
Source: J Med Internet Res. 2025 Oct 28;27:e78333. doi: 10.2196/78333 (PMC12605281; doi:10.2196/78333)
Supplement: Multimedia Appendix 1 [file jmir_v27i1e78333_app1.docx]

**(1). Self-introduction, Project Introduction, and Purpose Explanation**

Hello, I am XX, representing the Traditional Chinese Medicine Hospital in Yangzhou. I am a member of Director Dai's treatment team. This project conducting qualitative study to explore the experiences, facilitators, and barriers encountered during the implementation of the remote cancer symptom management with electronic patient‑reported outcome (ePRO) in China’s primary health care (PHC) settings. Our primary objective is to investigate the current landscape of cancer symptom management within PHC system, identify unmet needs among both PHC providers and community-dwelling cancer patients, and refine the electronic patient-reported outcome (ePRO) model. Through these efforts, we aim to reduce symptom burden, enhance functional recovery, and improve quality of life for the community-dwelling cancer patients. Would you be willing to participate in this interview?

**(2). Seeking Cooperation Intention, Signing the Informed Consent Form**

Thank you sincerely for your participation. We will be recording the entire interview process. If necessary, the interviewer will also take notes during the interview. All data will be kept strictly confidential and used solely for research purposes. If you agree to these terms, please carefully read the Informed Consent Form and sign it.

**(3). Completing the Personal Information Form**

Before the formal interview begins, please fill out the Personal Information Form. We will ensure confidentiality and use this information only for data analysis and statistics related to this research.

**(4). Commencing the Formal Interview and Recording According to the Interview Outline**

Thank you for your cooperation. We will now proceed with the formal interview and begin recording as per the interview outline.

**(5). Interview Conclusion, Checking the Completeness of Forms, and Verifying Key Content**

Our interview has concluded. Thank you very much for your active participation. Please ensure that the Informed Consent Form and Personal Information Form are complete and accurate. The information you have provided will be invaluable in enhancing the quality of management for community cancer patients. Wishing you and your family good health and happiness.
