# Supplementary material for: Implementation Challenges of Remote Cancer Symptom Management With Electronic Patient‑Reported Outcomes in China’s Primary Health Care Settings: Qualitative Study
Source: J Med Internet Res. 2025 Oct 28;27:e78333. doi: 10.2196/78333 (PMC12605281; doi:10.2196/78333)
Supplement: Multimedia Appendix 2 [file jmir_v27i1e78333_app2.docx]

**Community-dwelling cancer Patient**

**Ⅰ、Transition Questions**

(1). How long have you been diagnosed with cancer?

(2). What treatments have you received so far?

**Ⅱ、Core Questions**

(3). What has the follow-up management been like after your discharge? (Please specify where you received treatment, who is responsible for follow-up management, how it is managed, and provide evaluations on each aspect.)

(4). How do you feel about the current management? (Overall evaluation)

(5). What kind of management would you like to see in the future to better address your needs? (If the respondent's answers are not specific enough, ask follow-up questions. For example, which current management methods meet your needs, which do not, and how do you hope these issues can be addressed?)

(6). There is a new management model which you score health on your phone regularly. Your PHC providers gets your scores and takes care based on them. What do you think of this new model?

(7). What problems do you hope the new management model can solve for you?

(8). What concerns and worries do you have about the new management model?

**Ⅲ、Closing Questions**

(9). Do you have any other thoughts or suggestions regarding the topics we discussed today?

**Primary health care provider**

**Ⅰ. Transition Questions**

(1). How many years have you been involved in managing community-dwelling cancer patients? Approximately how many patients have you managed, and what cancer types do they have?

**Ⅱ. Core Questions**

(2). How do you evaluate the current management methods? What are the strengths and weaknesses?

(3). Do you think the PHC settings should provide substantial care for cancer patients, like they do for diabetes and hypertension?

(4). What suggestions do you have for improving the quality of management for community-dwelling cancer patients?

(5). There is a new management model, with a remote monitoring system where cancer patients self-report symptom scores via mobile app. When scores exceed clinical thresholds, PHC providers would receive alerts to initiate: (a) immediate interventions, or (b) specialist referrals if needed. What potential challenges do you foresee in implementing this model at PHC settings?

(6). To achieve better implementation, what conditions or equipment do you think are necessary? This could include guidance or suggestions from policies, tertiary hospitals, PHC settings, and individuals.

**Ⅲ. Closing Questions**

(7). Do you have any other thoughts or suggestions regarding the topics we discussed today?
