# Supplementary material for: Implementation Challenges of Remote Cancer Symptom Management With Electronic Patient‑Reported Outcomes in China’s Primary Health Care Settings: Qualitative Study
Source: J Med Internet Res. 2025 Oct 28;27:e78333. doi: 10.2196/78333 (PMC12605281; doi:10.2196/78333)
Supplement: Multimedia Appendix 3 [file jmir_v27i1e78333_app3.docx]

**Community-dwelling cancer Patient**

**Ⅰ. Transition Questions**

(1). Could you please share your experience with your cancer diagnosis and treatment?

**Ⅱ. Core Questions**

(2). How was your condition managed in the community health centres after you were discharged?

(3). Can you share your feelings and experiences with the current management model? (Overall evaluation)

(4). How would you like to see future treatment and management improved to better address your needs? (Which current management methods meet your needs, which do not, and how do you hope they can be improved?)

(5). What do you think you can do to help achieve better management and medical services?

(6). There is a new management model which you score health on your phone regularly. Your PHC providers gets your scores and takes care based on them. What do you think of this new model?

(7). What concerns and worries do you have about the new management model?

(8). Can you describe the support and care you receive from your family during your daily recovery?

(9). How has your work and social status changed before and after your illness?

**Ⅲ. Closing Questions**

(10). Do you have any other thoughts or suggestions regarding the topics we discussed today?

**Primary health care provider**

**Ⅰ. Transition Question**

(1). Could you share your work experience and experiences in managing the community-dwelling patients with cancers? For example, memorable events and interactions with cancer patients.

**Ⅱ. Core Questions**

(2). How do you evaluate the current management for cancer patients in your community? What achievements have been made, and what problems still exist?

(3). Do you think the PHC settings should provide substantial care for cancer patients, like they do for diabetes and hypertension?

(4). What suggestions do you have for improving the quality of management for community-dwelling cancer patients?

(5). There is a new management model, with a remote monitoring system where cancer patients self-report symptom scores via mobile app. When scores exceed clinical thresholds, PHC providers would receive alerts to initiate: (a) immediate interventions, or (b) specialist referrals if needed. What potential challenges do you foresee in implementing this model at PHC settings?

(6). To achieve better implementation, what conditions or equipment do you think are necessary? This could include guidance or suggestions from policies, tertiary hospitals, PHC settings, and individuals. (7). Can you discuss the cooperation between tertiary hospitals and PHC settings where you work? For example, how does bidirectional referral work in practice?

**Ⅲ、Ending Question**

(8). Do you have any other thoughts or suggestions regarding the topics we discussed today?
