# Supplementary material for: Implementation Challenges of Remote Cancer Symptom Management With Electronic Patient‑Reported Outcomes in China’s Primary Health Care Settings: Qualitative Study
Source: J Med Internet Res. 2025 Oct 28;27:e78333. doi: 10.2196/78333 (PMC12605281; doi:10.2196/78333)
Supplement: Multimedia Appendix 4 [file jmir_v27i1e78333_app4.docx]

**Medical supervisor**

Ⅰ. The member of the interviewer team presented the analysis results of in-depth interview.

(1). Current survival situation of community-dwelling cancer patients: The cancer patients often coexists with chronic diseases. However, they lacked cancer healthcare knowledge, encountered information barriers, and feel powerless in accessing digital healthcare channels. Patients experience heavy symptom burdens, withdraw from social interactions, and crave more effective management policies.

(2). Current status of cancer management in China’s PHC settings: Management policies are diverse but lack evaluation standards. Grassroots work primarily focuses on survival follow-up. Contact between tertiary hospitals is not close, and there is a lack of information data sharing. Health education lacks specificity, grassroots equipment is limited, and drug types are restricted. There is a shortage of special funds and insufficient human resources.

Ⅱ. Please allow experts present at the meeting to share their opinions and experiences in managing cancer or other chronic diseases based on the above content.

Ⅲ. In response to the above results, experts from various departments will discuss how to implement remote cancer symptom management with electronic patient‑reported outcome based on the aforementioned realities.

Ⅳ. Experts present at the meeting will discuss the advantages and disadvantages of mobile health, digital therapeutics, and tailored ePRO-based cancer symptom management.
