# Supplementary material for: Implementation Challenges of Remote Cancer Symptom Management With Electronic Patient‑Reported Outcomes in China’s Primary Health Care Settings: Qualitative Study
Source: J Med Internet Res. 2025 Oct 28;27:e78333. doi: 10.2196/78333 (PMC12605281; doi:10.2196/78333)
Supplement: Multimedia Appendix 5 [file jmir_v27i1e78333_app5.docx]

Identified facilitators and barriers in the 4 domains of the CFIR^a^ framework.

| Domain | | | Facilitators | Barriers | |
| --- | --- | --- | --- | --- | --- |
| I. Innovation | | | | | |
|  | A. Innovation source | | 1. The team has extensive experience with ePRO^b^ and is reliable (ePRO cancer symptom management). |  |  |
|  | B. Innovation evidence-based | | 2. The ePRO’s evidence base has been publicly published and peer-reviewed. |  |  |
|  | C. Innovation relative advantage | | 3. The senior medical professionals acknowledge its substantial advantages in cancer symptom management over the survival follow-up. |  |  |
|  | D. Innovation adaptability | | 4. The ePRO can be adapted to local conditions. |  |  |
|  | E. Innovation trialability | | 5. The ePRO can be piloted on a small scale. |  |  |
|  | F. Innovation complexity | |  |  | 1. The complexity of the regulatory scope of ePRO arises from its involvement in the 3-tiered health care system.  2. The complexity in the content and procedures (monitoring, alerting, intervening) of the ePRO. |
|  | G. Innovation design | | 6. The PRO with the digital function is well‑designed and simple to operate through an app. |  | 3. Older adult patients also have difficulty using electronic devices. |
|  | H. Innovation cost | | 7. Community-dwelling patients with cancer do not need to pay for the ePRO. |  | 4. The innovation requires financial, human, and material resource investments. |
| II. Outer setting | | | | | |
|  | A. Critical incidents | | N/A^c^ |  | N/A |
|  | B. Local attitudes | | 1. It is necessary to implement substantive management for the community-dwelling patients with cancer. |  | 1. The ePRO is not a mandated requirement led by the government. |
|  | C. Local conditions | | 2. The pilot regions are economically and politically above-average nationwide. |  | 2. Some rural areas have relatively underdeveloped economic conditions. |
|  | D. Partnerships and connections | | 3. The establishment of integrated health care networks, with upward‑upward-downward referral mechanism to empower the PHC^d^ providers to monitor-alert-intervene for the community-dwelling patients with cancer. |  | 3. The stability and quality of cooperation between the PHC system and tertiary hospitals need improvement, such as not sharing electronic medical record systems, leading to information barriers. |
|  | E. Policies and laws | | 4. The ePRO aligns with national policies such as “Healthy China 2030.” |  | 4. A lack of reference implementation procedures, manuals, and publicly available quality evaluation standards. |
|  | F. Financing | | 5. The ePRO project received 1 funding grant. |  | 5. The PHC system and patients also faced the funding shortage. |
|  | G. External pressure | | N/A |  | N/A |
| III. Inner setting | | | | | |
|  | A. Structural characteristics | |  |  |  |
|  |  | 1. Physical infrastructure |  |  | 1. The dispersed and complex nature of community environments. |
|  |  | 2. Information technology infrastructure | 1. Equipped with information technology devices such as internet access, computers, tablets, and smartphones. |  |  |
|  |  | 3. Work infrastructure | 2. The PHC team has formed the clinical care, public health, and management divisions. |  |  |
|  | B. Relational connections | |  |  | 2. Collaboration within the organization needs improvement. |
|  | C. Communications | | 3. Information sharing is achieved within the organization. |  |  |
|  | D. Culture | | 4. Human equality, and recipient and deliverer centeredness. |  | 3. There is room for improvement in the team’s learning culture. |
|  | E. Tension for change | | 5. Some PHC providers are willing to be the deliverer of the ePRO.  6. Most senior medical supervisors advocate for the implementation of the ePRO.  7. Community-dwelling patients with cancer expect the benefits brought by the ePRO. |  |  |
|  | F. Compatibility | |  |  | 4. Low compatibility with the existing workflows. |
|  | G. Relative priority | |  |  | 5. The implementation of ePRO has a lower priority than the “two diseases.” |
|  | H. Incentive systems | |  |  | 6. Unclear incentive mechanisms. |
|  | I. Mission alignment | |  |  | 7. PHC providers lacked clarity on the goal and meaning of the ePRO. |
|  | J. Available resources | |  |  |  |
|  |  | 1. Funding |  |  | 8. Limited funding. |
|  |  | 2. Space | N/A |  | N/A |
|  |  | 3. Materials and equipment |  |  | 9. Limited medical equipment and medications in the PHC settings. |
|  | K. Access to knowledge and information | |  |  | 10. Limited continuing medical education programs on specialized cancer knowledge and practical application are challenging for PHC providers.  11. Few health lectures at the grassroots level focus on cancer symptom management for community-dwelling patients with cancer. |
| IV. Individuals | | | | | |
|  | A. Need | | 1. The community-dwelling patients with cancer expect a better cancer care service. |  | 1. Low need among PHC providers for implementing the ePRO, with little benefit to them. |
|  | B. Capability | |  |  | 2. Inadequate knowledge and practical skills in cancer diagnosis and treatment among the primary health care personnel, especially village doctors, who have lower educational backgrounds.  3. Community-dwelling patients with cancer lack knowledge related to cancer symptom management, rehabilitation, and treatment. |
|  | C. Opportunity | | 2. With the government’s strong support for strengthening the PHC system, the opportunities for PHC providers will be improved. |  | 4. PHC providers have limited opportunities, as patients with cancer mistrust the PHC system and believe that it cannot provide much help, so they are unwilling to seek treatment at community health centers. |
|  | D. Motivation | |  | Low motivation among the PHC providers for implementing the ePRO | 5. Role ambiguity, they regard using ePRO to manage patients with cancer as the responsibility of tertiary hospital doctors.  6. Low self-efficacy, they with inadequate knowledge and outdated medical equipment are incapable of doing it. |
|  |  | |  | Low motivation among the community-dwelling patients with cancer to actively participate in the ePRO | 7. Stigma of illness, unwilling to let anyone know about their cancer situation.  8. Fatalism, leading to role avoidance and lack of active participation.  9. Shortage of money, afraid of increasing the financial burden on their family. |

^a^CFIR: Consolidated Framework for Implementation Research.

^b^ePRO: electronic patient-reported outcomes-based.

^c^N/A: Not applicable.

^d^PHC: primary health care.
