# Supplementary material for: Implementation Challenges of Remote Cancer Symptom Management With Electronic Patient‑Reported Outcomes in China’s Primary Health Care Settings: Qualitative Study
Source: J Med Internet Res. 2025 Oct 28;27:e78333. doi: 10.2196/78333 (PMC12605281; doi:10.2196/78333)
Supplement: Multimedia Appendix 6 [file jmir_v27i1e78333_app6.docx]

| ERIC Strategies | Cumulative Percent | Complexity | Design Quality & Packaging | Cost | Cosmopolitanism | External Policy & Incentives | Structural Characteristics | Networks & Communications | Culture | Tension for Change | Compatibility | Relative Priority | Organizational Incentives & Rewards | Goals and Feedback | Available Resources | Access to knowledge & information | Knowledge & Beliefs about the Intervention | Self-efficacy | Individual Stage of Change |
| --- | --- | --- | --- | --- | --- | --- | --- | --- | --- | --- | --- | --- | --- | --- | --- | --- | --- | --- | --- |
| Identify and prepare champions | **457%** | 30% | 15% | 12% | 15% | 22% | 27% | 17% | **52%** | 48% | 21% | 18% | 25% | 12% | 4% | 24% | 40% | 30% | 44% |
| Create a learning collaborative | **355%** | 33% | 7% | 8% | 31% | 15% | 18% | 35% | 30% | 9% | 14% | 4% | 13% | 12% | 9% | 45% | 16% | 30% | 28% |
| Alter incentive/allowance structures | **351%** | 7% | 0% | 44% | 0% | 41% | 18% | 0% | 15% | 22% | 10% | 39% | **71%** | 15% | 17% | 0% | 16% | 4% | 32% |
| Assess for readiness and identify barriers and facilitators | **349%** | 30% | 7% | 16% | 15% | 4% | 36% | 13% | 41% | 35% | 34% | 36% | 13% | 6% | 13% | 7% | 20% | 11% | 12% |
| Conduct educational meetings | **340%** | 13% | 22% | 12% | 12% | 15% | 5% | 13% | 22% | 17% | 10% | 7% | 0% | 21% | 0% | **79%** | **56%** | 15% | 20% |
| Capture and share local knowledge | **334%** | 27% | 15% | 4% | 23% | 26% | 23% | 26% | 22% | 13% | 14% | 14% | 8% | 12% | 22% | 31% | 24% | 19% | 12% |
| Conduct local consensus discussions | **332%** | 7% | 26% | 4% | 15% | 22% | 14% | 22% | 22% | 43% | 41% | 46% | 8% | 18% | 0% | 10% | 12% | 0% | 20% |
| Build a coalition | **316%** | 0% | 0% | 4% | **62%** | 33% | 27% | 39% | 19% | 9% | 21% | 18% | 17% | 15% | 17% | 3% | 16% | 0% | 16% |
| Promote adaptability | **309%** | 40% | 48% | 16% | 0% | 0% | 23% | 0% | 22% | 17% | 45% | 18% | 4% | 9% | 4% | 7% | 16% | 11% | 28% |
| Inform local opinion leaders | **297%** | 13% | 19% | 12% | 15% | 22% | 14% | 22% | 22% | 39% | 3% | 14% | 17% | 18% | 0% | 7% | 28% | 4% | 28% |
| Facilitation | **241%** | 20% | 7% | 8% | 12% | 4% | 9% | 26% | 30% | 0% | 24% | 14% | 4% | 18% | 4% | 10% | 20% | 22% | 8% |
| Access new funding | **240%** | 3% | 4% | **72%** | 4% | 7% | 5% | 4% | 0% | 0% | 3% | 11% | 38% | 3% | **78%** | 0% | 8% | 0% | 0% |
| Tailor strategies | **238%** | 27% | 15% | 12% | 0% | 11% | 18% | 4% | 30% | 13% | 38% | 14% | 17% | 0% | 9% | 0% | 12% | 11% | 8% |
| Develop a formal implementation blueprint | **235%** | 43% | 15% | 8% | 4% | 7% | 18% | 13% | 7% | 13% | 3% | 14% | 8% | 42% | 4% | 14% | 4% | 11% | 4% |
| Conduct local needs assessment | **228%** | 3% | 15% | 4% | 12% | 7% | 18% | 9% | 22% | 43% | 21% | 32% | 8% | 6% | 0% | 3% | 24% | 0% | 0% |
| Identify early adopters | **224%** | 20% | 11% | 8% | 4% | 7% | 23% | 17% | 11% | 13% | 10% | 7% | 13% | 6% | 0% | 10% | 20% | 19% | 24% |
| Conduct cyclical small tests of change | **215%** | 37% | 11% | 8% | 0% | 4% | 23% | 9% | 0% | 4% | 38% | 4% | 13% | 3% | 13% | 3% | 12% | 26% | 8% |
| Organize clinician implementation team meetings | **214%** | 20% | 4% | 0% | 0% | 0% | 14% | **52%** | 4% | 9% | 14% | 4% | 8% | 36% | 9% | 14% | 4% | 11% | 12% |
| Promote network weaving | **213%** | 0% | 0% | 0% | **50%** | 11% | 23% | **57%** | 11% | 4% | 0% | 4% | 8% | 6% | 9% | 10% | 12% | 4% | 4% |
| Develop educational materials | **211%** | 13% | 33% | 0% | 4% | 4% | 0% | 0% | 0% | 0% | 3% | 7% | 0% | 9% | 4% | **59%** | 36% | 19% | 20% |
| Audit and provide feedback | **198%** | 3% | 4% | 8% | 0% | 0% | 5% | 17% | 4% | 17% | 7% | 14% | 21% | **61%** | 0% | 3% | 4% | 22% | 8% |
| Conduct educational outreach visits | **193%** | 7% | 15% | 4% | 23% | 0% | 0% | 4% | 4% | 4% | 0% | 14% | 4% | 12% | 0% | 28% | 28% | 22% | 24% |
| Involve executive boards | **185%** | 0% | 4% | 20% | 23% | 41% | 14% | 9% | 19% | 13% | 3% | 11% | 13% | 0% | 17% | 0% | 0% | 0% | 0% |
| Conduct ongoing training | **184%** | 37% | 4% | 0% | 0% | 4% | 0% | 4% | 4% | 0% | 0% | 4% | 0% | 9% | 9% | 38% | 12% | 41% | 20% |
| Visit other sites | **177%** | 3% | 4% | 16% | 38% | 7% | 5% | 4% | 11% | 13% | 10% | 4% | 4% | 0% | 9% | 14% | 12% | 15% | 8% |
| Model and simulate change | **176%** | 27% | 11% | 20% | 8% | 4% | 14% | 4% | 7% | 13% | 3% | 0% | 0% | 9% | 0% | 7% | 4% | 33% | 12% |
| Fund and contract for clinical innovation | **176%** | 3% | 4% | 28% | 0% | 15% | 14% | 0% | 0% | 9% | 10% | 11% | 21% | 0% | 39% | 3% | 4% | 4% | 12% |
| Use advisory boards and workgroups | **171%** | 0% | 19% | 0% | 35% | 15% | 5% | 13% | 22% | 13% | 3% | 7% | 4% | 12% | 4% | 0% | 8% | 7% | 4% |
| Provide ongoing consultation | **171%** | 20% | 7% | 0% | 0% | 0% | 9% | 0% | 15% | 4% | 3% | 14% | 4% | 15% | 0% | 17% | 4% | 41% | 16% |
| Recruit, designate and train for leadership | **169%** | 7% | 0% | 4% | 15% | 0% | 18% | 17% | 33% | 4% | 0% | 11% | 21% | 18% | 4% | 3% | 4% | 4% | 4% |
| Stage implementation scale up | **155%** | 30% | 4% | 8% | 0% | 4% | 14% | 0% | 11% | 4% | 10% | 7% | 4% | 0% | 13% | 3% | 20% | 15% | 8% |
| Provide local technical assistance | **151%** | 17% | 4% | 4% | 4% | 7% | 18% | 9% | 0% | 0% | 14% | 0% | 0% | 12% | 0% | 24% | 0% | 22% | 16% |
| Involve patients/consumers and family members | **145%** | 0% | 19% | 0% | 4% | 11% | 9% | 9% | 11% | 22% | 10% | 18% | 4% | 9% | 0% | 3% | 0% | 4% | 12% |
| Make training dynamic | **142%** | 10% | 11% | 0% | 0% | 0% | 5% | 0% | 0% | 0% | 3% | 7% | 8% | 6% | 0% | 10% | 0% | 41% | 40% |
| Develop academic partnerships | **140%** | 0% | 4% | 4% | **50%** | 11% | 5% | 9% | 0% | 4% | 0% | 0% | 4% | 3% | 4% | 10% | 12% | 7% | 12% |
| Use an implementation adviser | **138%** | 10% | 15% | 4% | 8% | 4% | 5% | 9% | 4% | 13% | 10% | 7% | 0% | 12% | 13% | 14% | 0% | 7% | 4% |
| Develop and implement tools for quality monitoring | **135%** | 7% | 30% | 0% | 0% | 11% | 5% | 0% | 4% | 9% | 3% | 7% | 21% | 27% | 0% | 0% | 0% | 4% | 8% |
| Facilitate relay of clinical data to providers | **133%** | 3% | 7% | 0% | 0% | 4% | 0% | 4% | 4% | 22% | 3% | 7% | 4% | 36% | 0% | 10% | 12% | 7% | 8% |
| Distribute educational materials | **132%** | 3% | 19% | 0% | 0% | 0% | 0% | 4% | 4% | 13% | 0% | 4% | 0% | 3% | 0% | **55%** | 16% | 4% | 8% |
| Mandate change | **130%** | 7% | 0% | 8% | 0% | 15% | 5% | 4% | 11% | 13% | 3% | 32% | 13% | 0% | 0% | 0% | 4% | 4% | 12% |
| Purposely reexamine the implementation | **130%** | 17% | 22% | 0% | 4% | 11% | 0% | 4% | 7% | 4% | 28% | 4% | 4% | 12% | 4% | 0% | 4% | 0% | 4% |
| Develop resource sharing agreements | **117%** | 0% | 0% | 32% | 31% | 0% | 5% | 4% | 0% | 0% | 3% | 0% | 4% | 0% | 26% | 3% | 0% | 4% | 4% |
| Obtain and use patients/consumers and family feedback | **109%** | 0% | 30% | 4% | 0% | 0% | 5% | 0% | 7% | 9% | 10% | 7% | 13% | 9% | 0% | 0% | 4% | 4% | 8% |
| Obtain formal commitments | **107%** | 0% | 0% | 0% | 19% | 15% | 9% | 9% | 4% | 0% | 0% | 14% | 13% | 12% | 13% | 0% | 0% | 0% | 0% |
| Increase demand | **105%** | 3% | 4% | 12% | 0% | 0% | 0% | 0% | 4% | 13% | 0% | 29% | 8% | 0% | 4% | 0% | 20% | 0% | 8% |
| Shadow other experts | **100%** | 7% | 4% | 0% | 4% | 0% | 5% | 0% | 7% | 4% | 3% | 0% | 0% | 0% | 0% | 21% | 4% | 33% | 8% |
| Change physical structure and equipment | **98%** | 3% | 0% | 4% | 0% | 0% | 32% | 0% | 0% | 0% | 7% | 0% | 4% | 0% | 48% | 0% | 0% | 0% | 0% |
| Place innovation on fee for service lists/formularies | **91%** | 0% | 0% | 24% | 0% | 19% | 0% | 0% | 0% | 4% | 3% | 7% | 8% | 0% | 17% | 0% | 0% | 0% | 8% |
| Use other payment schemes | **89%** | 0% | 0% | 20% | 0% | 15% | 0% | 0% | 0% | 0% | 0% | 7% | 25% | 0% | 22% | 0% | 0% | 0% | 0% |
| Centralize technical assistance | **86%** | 10% | 4% | 0% | 4% | 0% | 5% | 26% | 4% | 0% | 10% | 0% | 0% | 9% | 0% | 3% | 0% | 11% | 0% |
| Provide clinical supervision | **85%** | 7% | 0% | 0% | 0% | 0% | 0% | 4% | 4% | 0% | 10% | 0% | 8% | 15% | 0% | 17% | 0% | 11% | 8% |
| Develop and organize quality monitoring systems | **81%** | 10% | 0% | 4% | 0% | 15% | 5% | 0% | 0% | 9% | 3% | 0% | 4% | 24% | 0% | 0% | 0% | 7% | 0% |
| Use train the trainer strategies | **80%** | 7% | 0% | 0% | 8% | 4% | 0% | 9% | 0% | 0% | 0% | 0% | 4% | 3% | 9% | 10% | 4% | 15% | 8% |
| Create or change credentialing and/or licensure standards | **79%** | 0% | 0% | 4% | 4% | 19% | 5% | 0% | 0% | 4% | 0% | 14% | 17% | 0% | 4% | 0% | 0% | 0% | 8% |
| Work with educational institutions | **78%** | 0% | 11% | 4% | 19% | 4% | 5% | 4% | 4% | 0% | 3% | 0% | 4% | 0% | 4% | 7% | 4% | 0% | 4% |
| Make billing easier | **76%** | 3% | 0% | 32% | 0% | 7% | 0% | 0% | 0% | 0% | 0% | 4% | 8% | 0% | 22% | 0% | 0% | 0% | 0% |
| Revise professional roles | **73%** | 3% | 0% | 0% | 0% | 4% | 18% | 0% | 4% | 0% | 10% | 0% | 13% | 9% | 9% | 3% | 0% | 0% | 0% |
| Develop disincentives | **67%** | 0% | 0% | 16% | 0% | 7% | 5% | 0% | 4% | 9% | 0% | 7% | 8% | 3% | 0% | 0% | 0% | 0% | 8% |
| Use data experts | **66%** | 3% | 4% | 12% | 4% | 4% | 0% | 4% | 0% | 17% | 0% | 7% | 0% | 3% | 0% | 0% | 4% | 4% | 0% |
| Use mass media | **62%** | 0% | 0% | 4% | 8% | 15% | 0% | 9% | 0% | 4% | 3% | 7% | 0% | 0% | 0% | 3% | 8% | 0% | 0% |
| Change record system | **54%** | 0% | 0% | 0% | 4% | 4% | 9% | 9% | 0% | 4% | 0% | 7% | 0% | 9% | 4% | 0% | 0% | 4% | 0% |
| Alter patient/consumer fees | **53%** | 0% | 0% | 20% | 0% | 7% | 0% | 0% | 0% | 0% | 0% | 0% | 4% | 0% | 22% | 0% | 0% | 0% | 0% |
| Start a dissemination organization | 48% | 0% | 4% | 0% | 8% | 0% | 9% | 13% | 7% | 0% | 0% | 0% | 0% | 3% | 0% | 0% | 4% | 0% | 0% |
| Create new clinical teams | 48% | 3% | 0% | 0% | 0% | 0% | 9% | 13% | 4% | 0% | 7% | 0% | 0% | 0% | 4% | 0% | 0% | 7% | 0% |
| Develop an implementation glossary | 48% | 3% | 4% | 4% | 4% | 4% | 9% | 0% | 0% | 0% | 0% | 0% | 0% | 9% | 0% | 7% | 4% | 0% | 0% |
| Change accreditation or membership reqs | 47% | 0% | 0% | 4% | 8% | 15% | 5% | 0% | 0% | 0% | 0% | 4% | 8% | 0% | 0% | 0% | 0% | 0% | 4% |
| Intervene with patients/consumers to enhance uptake & adherence | 41% | 3% | 7% | 4% | 0% | 4% | 0% | 0% | 4% | 0% | 3% | 4% | 4% | 0% | 0% | 0% | 0% | 0% | 8% |
| Change liability laws | 38% | 0% | 0% | 0% | 0% | 19% | 0% | 0% | 0% | 4% | 7% | 0% | 8% | 0% | 0% | 0% | 0% | 0% | 0% |
| Use capitated payments | 32% | 0% | 0% | 16% | 0% | 0% | 0% | 0% | 0% | 0% | 0% | 4% | 4% | 0% | 9% | 0% | 0% | 0% | 0% |
| Prepare patients/consumers to be active participants | 27% | 0% | 0% | 0% | 0% | 0% | 0% | 0% | 7% | 9% | 3% | 0% | 4% | 3% | 0% | 0% | 0% | 0% | 0% |
| Change service sites | 25% | 0% | 0% | 0% | 0% | 4% | 14% | 0% | 0% | 0% | 3% | 0% | 0% | 0% | 4% | 0% | 0% | 0% | 0% |
| Remind clinicians | 18% | 0% | 0% | 0% | 0% | 0% | 0% | 4% | 0% | 0% | 0% | 4% | 0% | 3% | 0% | 3% | 0% | 0% | 4% |
| Use data warehousing techniques | 0% | 0% | 0% | 0% | 0% | 0% | 0% | 0% | 0% | 0% | 0% | 0% | 0% | 0% | 0% | 0% | 0% | 0% | 0% |

Note: CFIR, Consolidated Framework for Implementation Research; ERIC, Expert Recommendations for Implementing Change
