# Supplementary material for: Implementation Challenges of Remote Cancer Symptom Management With Electronic Patient‑Reported Outcomes in China’s Primary Health Care Settings: Qualitative Study
Source: J Med Internet Res. 2025 Oct 28;27:e78333. doi: 10.2196/78333 (PMC12605281; doi:10.2196/78333)
Supplement: Multimedia Appendix 7 [file jmir_v27i1e78333_app7.docx]

| CFIR-ERIC^a^ matched original strategies | Detailed explanation of the original strategies | Expert-discussed revised strategies (first round) | Expert-discussed revised strategies (second round) | Simplifying strategies for our specific context |
| --- | --- | --- | --- | --- |
| Identify and prepare champions | Identify and prepare individuals who dedicate themselves to supporting, marketing, and driving through an implementation, overcoming indifference or resistance that the intervention may provoke in an organization. | Engage specific primary health care personnel to develop a comprehensive understanding of and commitment to the ePRO^b^ project. | Prepare multi-stakeholders to dedicate themselves to supporting and driving the implementation of ePRO by advocating governmental policy guidelines and demonstrating evidence-based best practices. | Engaged EBP^c^ stakeholders |
| Create a learning collaborative | Facilitate the formation of groups of providers or provider organizations and foster a collaborative learning environment to improve the implementation of the clinical innovation. | Convene regular internal learning and discussion sessions among organizational members. | Monthly intradepartmental knowledge-sharing sessions featuring: case-based presentations; critical appraisal of peer-reviewed literature relative to ePRO. | Peer-led ePRO sessions |
| Alter incentive or allowance structures | Work to incentivize the adoption and implementation of the clinical innovation. | Establish appropriate incentive mechanisms, both financial and nonfinancial. | Receipt of practice remuneration for any verified time clinicians spend on the ePRO management (eg, preparing materials for a session or follow-up). | EBP incentives |
| Assess for readiness and identify barriers and facilitators | Assess various aspects of an organization to determine its degree of readiness to implement, barriers that may impede implementation, and strengths that can be used in the implementation effort. | Assess the operational realities of primary hospitals and formulate targeted solutions to address identified issues. | On-site assessments of primary care facilities to evaluate challenges in ePRO implementation and formulate resolution strategies were discussed with a multidisciplinary expert task force. | Field-informed expert solutions for ePRO |
| Conduct educational meetings | Hold meetings targeted toward different stakeholder groups (eg, providers, administrators, other organizational stakeholders, and community, patient or consumer, and family stakeholders) to teach them about the clinical innovation. | Organize training sessions to facilitate understanding of the ePRO symptom management system and impart required operational skills. | Held one-on-one instruction and group training sessions to enhance both health care providers’ and patients’ understanding of the electronic symptom management system. | Training for the ePRO system |
| Build a coalition | Recruit and cultivate relationships with partners in the implementation effort. | Establish a hierarchical diagnosis and treatment framework for symptom management in oncology patients. | Develop a hierarchical symptom monitoring framework for oncology patients, drawing on proven community health strategies for chronic disease management. | Develop tiered cancer management through ePRO |
| Access new funding | Access new or existing money to facilitate the implementation. | Secure and allocate research grants to ensure sustained project implementation. | The project leadership coordinates with primary care facilities to submit competitive proposals for government-sponsored research grants (national or provincial tiers) | Access new funding |
| Organize clinician implementation team meetings | Develop and support teams of clinicians who are implementing the innovation and give them protected time to reflect on the implementation effort, share lessons learned, and support one another’s learning. | Hold regular meetings among primary health care personnel to discuss implementation challenges of the ePRO system and exchange practical experiences. | The project team facilitates monthly structured problem-solving sessions with primary care clinicians to support them by implementing ePRO. | Expert-led ePRO consultation |
| Promote network weaving | Identify and build on existing high-quality working relationships and networks within and outside the organization, organizational units, teams, etc, to promote information sharing, collaborative problem-solving, and a shared vision or goal related to implementing the innovation. | Forge a medical alliance between primary, county-level, and higher hospitals. | Build on existing high-quality working relationships and networks across primary care hospitals, secondary hospitals, and tertiary hospitals. | Establish clinical support partnerships |
| Develop educational materials | Develop and format manuals, toolkits, and other supporting materials in ways that make it easier for stakeholders to learn about the innovation and for clinicians to learn how to deliver the clinical innovation. | Produce clinical pathways, instructional videos, and educational manuals. | Developed manuals, toolkits, and other supporting materials to make it easier for stakeholders to learn about the ePRO monitoring. | Develop ePRO-related toolkits |
| Audit and provide feedback | Collect and summarize clinical performance data over a specified period and give it to clinicians and administrators to monitor, evaluate, and modify provider behavior. | Conduct regular analysis and reporting of clinical monitoring data for clinicians and administrators. | Conduct monthly quality control assessments covering: follow-up adherence rates, longitudinal symptom trajectory data, and triggered clinical alerts. | Audits with performance feedback |
| Develop academic partnerships | Partner with a university or academic unit for shared training and bringing research skills to an implementation project. | Collaborate with universities or research institutes to address methodological and informatics challenges. | Tertiary-academic partnerships (tertiary hospitals or academic institutions) deliver national oncology continuing medical education programs to primary care teams. | EBP-based continuing medical education |
| Distribute educational materials | Distribute educational materials (including guidelines, manuals, and toolkits) in person, by mail, or electronically. | Disseminate clinical pathways, instructional videos, and educational manuals. | Community-based participatory health promotion using mixed-media education (printed booklets and videos) via free medical consultation camps and public health literacy lectures. | Disseminate paper or web-based educational materials |

^a^CFIR-ERIC: Consolidated Framework for Implementation Research–Expert Recommendations for Implementing Change.

^b^ePRO: electronic patient-reported outcomes.

^c^EBP: engage evidence-based practices.
